# Inside a mystery of oncoscience: The cancer-sniffing pets

**DOI:** 10.18632/oncoscience.490

**Published:** 2019-09-03

**Authors:** Luca Roncati

**Affiliations:** ^1^ Institute of Pathology, University Hospital of Modena, Policlinico, Modena, Italy

**Keywords:** cancer, necrosis, cadaverine, putrescine, pets

**Figure 1:**
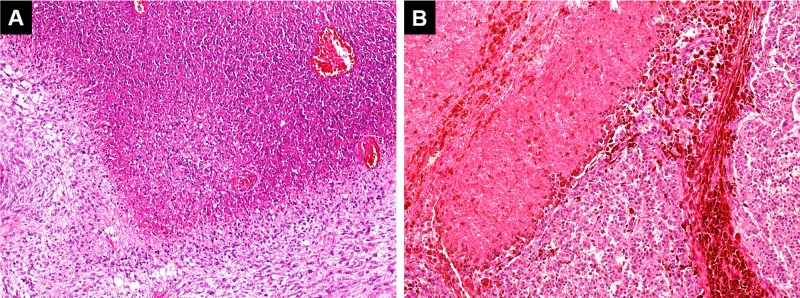
Pseudopalisading necrosis in an IDH-wildtype glioblastoma which occurred in the right temporal lobe of a 50-year-old female patient, recently diagnosed by the author [A: hematoxylin & eosin, 10x] Significantly, members of the patient's family reported that their cat, which had successfully undergone veterinary surgery for a necrotic skin melanoma one year earlier [B: hematoxylin & eosin, 10x], displayed bizarre behavior when the patient (its owner) began to manifest symptoms of the disease. The cat's bizarre behavior, similar to that observed when it was sick, might well be explained as a conditioned response to its own necrotic tumor (now healed) triggered by the perception of necrosis in its owner. However, it should not be excluded that such behavior might have been due to the cat's innate ability to detect necrosis, and would have been displayed even if it had not previously developed a necrotic cancer.

Worldwide, sick people are daily enjoying the benefits of pet-therapy [1–6]. Next to this scientific evidence, the media report cases of patients who claim to have been saved by their cancer-sniffing pets through an early diagnosis of malignancy. By virtue of this, the concept of ‘canine cancer detection’ has been advanced, on the basis of the presumed olfactory ability of pets, in particular dogs, to smell very low concentrations of aromatic and/or alkanes compounds generated and released by malignant tumors in the patient's breath, urine or watery stool and into adsorbent materials [7–15]. It is well known that the brain of a domestic dog (*Canis lupus familiaris* from the Latin) is dominated by a wide olfactory cortex unlike the humans, where a visual cortex predominates. More in detail, dogs are equipped up to 56 times more smell-sensitive receptors than the human beings, reaching the number of 280 million in selected breeds, spread over an olfactory surface about the size of a pendrive (9.76 cm^2^), if compared to 5 million over an area about the size of a postage stamp (3.08 cm^2^) for the humans [16, 17]. This is thought to render its sense of smell up to 56 times more sensitive than human's. The domestic cat (*Felis silvestris catus* from the Latin) also possesses an acute sense of smell, due to its well-developed olfactory bulb and, in addition, to a large surface of olfactory mucosa (about 5.8 cm^2^), which is almost twice that of the human beings [16]. In oncological medicine, among the diagnostic hallmarks of malignancy there are: lymphovascular and perineural invasion; infiltrative neoplastic growth; immune evasion; a high cytoproliferative index; an elevated mitotic cell count; and tumor necrosis [18, 19]. More in detail, tumor necrosis (νέκρωσις – death from the Greek) is a form of hypoxic death related to the high metabolic demand of cancer cells. It does not follow the apoptotic cascade, but the uncontrolled release of cell death products evokes in the surrounding space an inflammatory response which attracts leukocytes, resulting in an accumulation of cell debris and decomposing dead tissue [20, 21]. It is notorious that the decomposition process produces foul-smelling toxic diamines, such as pentamethylenediamine (cadaverine) and tetramethylenediamine (putrescine), which are the main source of the putrid odor in decaying animal tissue [22–27]. Therefore, it is possible that some pets, more likely some dogs, are able to detect the odor of tumor necrosis, deriving from aggressive neoplasms, in their owners or on themselves (Figure 1), thanks to their extraordinary sense of smell. Obviously, a tumor developed near to the skin surface should be easier to detect than one deeply located. In this regard, even the modern nanotechnologies seem to support the possibility to sniff cancer, since sophisticated olfactory sensors have been patented for diagnostic purposes on the humans with amazing results [28–32]. However, it is unlikely that this pet ability can be exploited in cancer screening models, because the pet realistically requires enough time to set the normal odor status of its owner, in order to be able to capture the slightest odor changes in the future domestic partnership. The use of trained molecular dogs would be also affected by errors due to various non-neoplastic morbid conditions with superimposed necrosis, such as that from phlegmons, abscesses or gangrene. In spite of these reservations, there are all the prerequisites to consider rudimental “pet-diagnosis” a scientific fact.
